# A cross-sectional survey of compliance with national guidance for alcohol consumption by children: measuring risk factors, protective factors and social norms for excessive and unsupervised drinking

**DOI:** 10.1186/1471-2458-10-547

**Published:** 2010-09-10

**Authors:** Mark A Bellis, Michela Morleo, Karen Hughes, Jennifer Downing, Sara Wood, Linda Smallthwaite, Penny A Cook

**Affiliations:** 1 Centre for Public Health, Liverpool John Moores University, Henry Cotton Campus, 15-21 Webster Street, Liverpool, L3 2ET, UK; 2 Warrington & Halton Trading Standards Service, Warrington Borough Council, Public Protection Services, Business Support Centre, New Town House, Buttermarket Street, Warrington, WA1 2NH, UK

## Abstract

**Background:**

The Chief Medical Officer for England has developed the first guidance in England and some of the first internationally on alcohol consumption by children. Using the most recent iteration of a large biennial survey of schoolchildren we measure the extent to which young people's drinking fell within the guidelines just prior to their introduction and the characteristics of individuals whose drinking does not; how alcohol related harms relate to compliance; and risk factors associated with behaving outside of the guidance.

**Methods:**

A cross-sectional survey was conducted utilising a self-completed questionnaire with closed questions. A total of 11,879 schoolchildren, aged 15-16 years, from secondary schools in North West England participated in the study. Data were analysed using chi square and conditional logistic regression.

**Results:**

Alcohol consumption is an established norm by age 15 years (81.3%). Acute alcohol related violence, regretted sex and forgetfulness were experienced by significantly fewer children drinking within the guidance (than outside of it). Over half of drinkers (54.7%) reported routinely drinking more heavily than guidance suggests (here ≥5 drinks/session ≥1 month), or typically drinking unsupervised at home or at a friend's home when parents were absent (57.4%). Both behaviours were common across all deprivation strata. Children with greater expendable incomes were less likely to consume within guidance and reported higher measures for unsupervised, frequent and heavy drinking. Although drinking due to peer pressure was associated with some measures of unsupervised drinking, those reporting that they drank out of boredom were more likely to report risk-related drinking behaviours outside of the guidance.

**Conclusions:**

Successful implementation of guidance on alcohol consumption for children could result in substantial reductions in existing levels of alcohol related harms to young people. However, prolonged social marketing, educational and parental interventions will be required to challenge established social norms in heavy and unsupervised child drinking across all social strata. Policy measures to establish a minimum price for alcohol and provide children with entertaining alternatives to alcohol should also increase compliance with guidance.

## Background

Worldwide, alcohol misuse is estimated to be responsible for 4.0% of the global burden of disease [1]. It has strong associations with health, social and economic harms [2,3], and young people are particularly vulnerable to such harms [4]. In particular, adolescence is a time of rapid brain maturation between childhood and adulthood resulting in structural and functional changes [5]. Heavy alcohol consumption during adolescence has been associated with the disruption of brain development [6-8]. Furthermore, young people are more likely to be naive drinkers and less able to cope with inebriation [9,10]; are likely to binge by consuming large amounts in a small time [11]; and may also consume alcohol in places that may leave them exposed to other harms (e.g. covertly in parks [12]). In the UK, it is illegal for a child to be given alcohol under the age of five. At age 16, legislation permits alcohol consumption in restaurants and some pubs as part of a table meal and under adult supervision. The legal age of independent drinking and alcohol purchase in public settings is 18 [13]. With high levels of youth alcohol misuse and associated harms in the UK [14], along with no consensus on if, when or how children should be introduced to alcohol, the Chief Medical Officer (CMO) for England developed national guidance on alcohol consumption by young people under the age of legal purchase [4]. Relatively few other countries have taken such a step with many leaving levels entirely up to parental discretion and others identifying an age below which any alcohol consumption is illegal regardless of location or supervision (e.g. Japan) [15]. On the occasions where countries have provided guidance on alcohol consumption it can range from simple statements on age of consumption (e.g. Republic of Ireland [16]), through advice to prolong abstinence in those who have not begun drinking (e.g. the Netherlands [17]) to more detailed guidance on age and levels of consumption (e.g. Australia [18]).

With little established and evaluated practice elsewhere, the English guidance was developed largely from a systematic review of literature on alcohol consumption and related harms and expert opinion, followed by a wide public consultation on how best to communicate the CMO guidance in order to help people make informed and sensible decisions (29^th ^January to 23^rd ^April, 2009) [4]. The final guidance on consumption included: that an alcohol free childhood is the best option; that onset of drinking should be delayed as long as possible and at least until 15 years old; and that if 15-17 year olds do drink, that they should do so only under the supervision or guidance of a parent or carer, should not drink more than once a week, and should never exceed the recommended maximum daily units for adults (females: 2-3 units; males: 3-4 units; one unit = 8 g pure alcohol). The guidance also stressed that on days when they drink consumption should usually be below such levels. The final guidance was released on 17th December 2009. As advisory guidance this required no legislative changes to English law but aimed to establish a professional and evidence based position on underage alcohol consumption that empowers parents in discussions with children [4]. Moreover, while not legally enforceable such guidance for parents, professionals and young people could contribute to and help re-enforce strategies to reduce alcohol misuse by young people. However, its effectiveness and consequent utility requires an understanding of how much the guidance differs from established drinking patterns (i.e. social norms) and the potential harms avoided by changing the consumption of aberrant individuals.

The North West region of England experiences disproportionately high levels of alcohol misuse and related harms [19]. Consequently, here we use a large established biennial survey conducted just prior to implementation of the guidance to examine the extent to which young people's drinking already fell within the guidelines, the characteristics of those individuals whose drinking does not, and how alcohol related harms relate to compliance. From a range of potential risk and protective factors, we examine which are most strongly associated with behaving outside of the guidance and how such associations may inform public health interventions.

## Methods

An anonymous cross-sectional self-completed school survey was conducted in 2009 to investigate drinking patterns among young people resident in the region. The survey is biennial (from 2005) with this iteration being delivered between March and April 2009 in 21 of 22 North West Trading Standards (local authority) areas. In England local authorities are sub-regional administrative areas governed by a locally elected council. The questionnaire consists of closed, self-completed questions which addressed young people's current drinking behaviour. Questions included: demographics (age, sex and postcode of residence); usual frequency of alcohol consumption and bingeing (here, heavy drinking - drinking five or more drinks in one session [14]); types of alcohol products consumed in a typical week (e.g. cans of beer, bottles of wine) and how individuals access alcohol (e.g. buy themselves, parents provide knowingly, take from parents without consent, etc). Individuals were also asked to identify where they mostly drink alcohol (at home, outside in parks, streets and around shops, etc) including whether their parents are present when they are drinking. To measure the impact of consumption, respondents identified whether they: had ever been violent or in a fight whilst drunk; had regretted having had sex with someone after drinking; and tended to forget things when they had been drinking alcohol. Individuals also provided details of their weekly expendable income and whether they were involved in hobbies or sports activities out of school. Finally, questions also addressed why they drank; specifically asking if they drink alcohol because their friends do (i.e. peer pressure) and/or because there is nothing else to do (i.e. boredom).

The questionnaire was made available to secondary schools across the North West for whom participation was voluntary. Students were informed that participation was voluntary and anonymous and that data were collected solely for the purpose of aggregated analyses. All aspects of the research methodology complied fully with the Helsinki Declaration. The survey was established by Local Authority Trading Standards in the North West and was scrutinised and approved by the Trading Standards North West Executive committee and supported by the cross-departmental Alcohol Forum at Government Office North West. Formal ethical approval was not requested in 2009 as this survey is an ongoing biennial process established by Trading Standards in 2005 (in agreement with public sector partners) as an audit of their role in preventing alcohol sales to minors [20]. School staff delivered questionnaires to students in years 10 and 11 (i.e. ages 14 to 17 years) within normal school hours [21]. Previous North West surveys of youth alcohol consumption in this series [20,21] provided appropriate sample sizes (target 10,000). Sampling was completed after a total of 133 public and private schools (21% of North West total of 620) across 21 local authority areas had participated, providing 13,903 completed questionnaires. For comparability with other major surveys of youth drinking (e.g. European School Survey Project on Alcohol and other Drugs, ESPAD [14]), the sample was then restricted to those aged 15 or 16 for analysis (n = 11,879). Response rates were not recorded in each class as the sample was not intended to be representative but opportunistic for both students and classroom participation. Analyses focus on relationships between variables recorded by individual participants and across ecological categories of deprivation.

In order to calculate level of deprivation, postcode of residence was mapped to a Lower Super Output Area (LSOA), small geographical areas with an average population of approximately 1,500 [22]. All LSOAs in England have an Index of Multiple Deprivation value, where a higher score indicates a higher level of deprivation. Where individual postcode was unavailable (n = 3,166; 26.7%), based on a methodology validated elsewhere [20,21,23], school postcode was used as a proxy. From all variables collected, eight proxy measures were used to assess whether respondents' behaviour fell within the CMO guidance (Table 1).

**Table 1:** Proxy survey measures relating to guidance on alcohol consumption by children

CMO Guidance	Proxy measure assessed	Short description
**Drink at all**	**Ever drink alcohol**	**Drink**

Unsupervised drinking or drinking without parental guidance	Drink at home or at friend's home when parents are out^$^	Unsupervised inside
	Drink outside in shops, parks and streets^$^	Unsupervised outside
	Buy own alcohol	Self-purchase
	Take alcohol from parents without permission	Take from parents
	Ask adults outside shops to buy them alcohol	Proxy purchase

Excessive drinking	Reported drinking frequency >1 session/week	Frequent drinking
	≥5 drinks/session (bingeing) ≥1 per month	Heavy drinking

^$^Questions asked where individuals mostly drink. Respondents could tick multiple answers.

Analyses used chi square and backward conditional logistic regression (CLR) in SPSS v17 with stratification by local authority of school included in CLR analyses to identify and control for sub population effects. The final sample represents 6.5% of all of 15-16 year olds in the North West region and response rates for individual questions are identified in Table 2.

**Table 2:** Characteristics of youths drinking alcohol at all or in ways that contravene national consumption guidance

	***All***		***Drinkers only***							
				***Unsupervised consumption and access***					***Excess drinking***	
				
	**n**	***% drink alcohol***	**n**	**% drink unsupervised inside**	**% drink unsupervised outside**	**% self-purchase**	**% take from parents**	**% proxy purchase**	**% drink frequently**^**§**^	**% drink heavily**^**§**^

Age										
15	5955	81.3	4834	55.4	30.1	21.0	8.7	12.6	20.8	51.0
16	5924	83.3	4932	59.3	29.5	29.9	8.3	11.2	23.9	58.4
P		0.004		<0.001	0.481	<0.001	0.460	0.026	<0.001	<0.001

Sex										
Female	5959	85.9	5118	61.3	28.6	25.8	9.1	10.5	20.2	54.4
Male	5920	78.7	4648	53.0	31.1	25.2	7.9	13.5	24.8	55.0
P		<0.001		<0.001	0.007	0.523	0.033	<0.001	<0.001	0.549

Deprivation (IMD^ Score)										
< = 10 (affluent)	2686	86.6	2323	60.9	28.6	23.6	10.8	9.2	21.9	53.7
>10-20	2731	85.5	2331	57.5	28.7	25.2	8.0	10.2	20.2	51.9
>20-30	1608	82.7	1330	57.8	29.6	24.0	8.5	11.5	22.6	53.4
>30-40	1404	80.7	1131	54.8	29.9	28.5	5.7	12.7	20.9	56.7
>40 (poor)	3213	78.2	2510	54.8	31.8	27.3	8.2	15.2	25.3	57.8
P		<0.001		<0.001	0.113	0.005	<0.001	<0.001	<0.001	<0.001

Expendable income										
< = £10	5722	77.5	4400	51.3	26.4	18.1	7.9	10.6	17.5	46.4
£11-20	2864	83.9	2392	61.8	31.3	24.1	8.5	12.3	22.7	56.8
£21-30	1359	87.3	1180	61.4	30.7	33.3	9.2	12.3	26.0	61.3
>£30	1927	90.7	1741	63.9	35.7	40.4	9.5	14.2	31.6	68.2
P		<0.001		<0.001	<0.001	<0.001	0.160	<0.001	<0.001	<0.001

Hobby/Sport										
No	3316	81.1	3314	58.5	34.4	26.0	8.0	13.5	25.1	59.8
Yes	6287	82.9	6277	56.8	27.1	25.2	8.7	10.9	20.9	51.8
P		0.014		0.118	<0.001	0.389	0.235	<0.001	<0.001	<0.001

Parents provide										
No			4805	59.4	40.1	32.7	8.3	17.7	26.1	63.3
Yes			4734	56.3	19.8	18.2	8.7	6.0	19.0	47.1
P				0.002	<0.001	<0.001	0.436	<0.001	<0.001	<0.001

Drinking Pressures										
Peer pressure No			7189	54.6	27.1	24.9	7.6	10.4	22.0	53.1
Peer pressure Yes			2358	66.6	38.0	27.5	11.3	15.7	23.5	59.9
P				<0.001	<0.001	0.011	<0.001	<0.001	0.136	<0.001
Boredom No			7167	55.5	21.7	23.1	7.1	7.6	17.7	48.1
Boredom Yes			2352	64.4	54.6	32.9	13.0	24.06	36.9	75.2
P				<0.001	<0.001	<0.001	<0.001	<0.001	<0.001	<0.001

Alcohol related harm										
Violence No			6940	54.4	21.8	19.3	7.5	7.9	14.6	45.9
Violence Yes			2375	66.9	53.1	44.0	11.2	23.2	45.8	80.9
P				<0.001	<0.001	<0.001	<0.001	<0.001	<0.001	<0.001
Regretted sex No			8229	56.0	27.3	23.0	8.0	10.5	19.6	51.3
Regretted sex Yes			988	69.0	48.9	46.4	12.6	21.5	45.1	81.2
P				<0.001	<0.001	<0.001	<0.001	<0.001	<0.001	<0.001
Forget things No			5445	50.9	22.9	21.6	6.5	8.5	18.1	44.9
Forget things Yes			3995	67.4	39.6	30.9	11.3	16.2	28.5	69.0
P				<0.001	<0.001	<0.001	<0.001	<0.001	<0.001	<0.001

^§ ^drinking frequently = >1 session/week; drinking heavily = drink ≥5 drinks/session ≥1 per month; ^IMD = Index of multiple deprivation.

## Results

Overall, 82.3% of participants reported that they consumed alcohol. The vast majority of respondents were already drinking at age 15 (81.3%) with higher levels in females and those from more affluent areas (Tables 2 &3). Individuals with higher personal income and those involved in a hobby/sport were also more likely to drink (Tables 2 &3).

**Table 3:** Conditional logistic regression analyses predicting drinking, accessing alcohol without supervision and engaging in risky drinking practices

		***All***	***Drinkers only***						
		
			***Unsupervised consumption and access***					***Excess drinking***	
			
		***Drink alcohol***	Unsupervised inside drinking	Outside drinking (streets, parks, shops)	Self-purchase	Take from parents	Proxy purchase	**Frequent drinking**^**§**^	**Heavy drinking**^**§**^
n (% yes)		11,879 (82.3%)	9,720 (57.4%)	9,720 (29.8%)	9,549 (25.5%)	9,549 (8.5%)	9,549 (11.9%)	9,766 (22.4%)	9,664 (54.7%)

		AOR (95%CI)	AOR (95%CI)	AOR (95%CI)	AOR (95%CI)	AOR (95%CI)	AOR (95%CI)	AOR (95%CI)	AOR (95%CI)

Age	15	Ref*	Ref*	ns	Ref***	ns	Ref*	Ref*	Ref***
	16	1.13(1.02-1.25)	1.11(1.01-1.21)		1.51(1.37-1.67)		0.83(0.73-0.96)	1.12(1.01-1.24)	1.29 (1.18-1.41)

Sex	Female	Ref***	Ref***	Ref***	ns	Ref**	Ref***	Ref***	Ref*
	Male	0.55(0.49-0.61)	0.69(0.63-0.75)	1.23(1.11-1.36)		0.80(0.68-0.93)	1.36(1.18-1.56)	1.43(1.28-1.59)	1.11(1.02-1.22)

Deprivation IMD^ score	< = 10 (affluent)	Ref***	Ref**	ns	ns	Ref**	Ref**	Ref**	ns
	>10-20	0.92(0.78-1.08)	0.88(0.78-1.00)			0.79(0.64-0.98)	1.04(0.84-1.30)	0.93(0.79-1.08)	
	>20-30	0.73(0.61-0.88)	0.89(0.76-1.04)			0.87(0.674-1.12)	1.13(0.88-1.45)	1.05(0.87-1.25)	
	>30-40	0.65(0.54-0.78)	0.75(0.63-0.88)			0.53(0.39-0.73)	1.28(0.99-1.66)	0.90(0.74-1.09)	
	>40 (poor)	0.57(0.48-0.66)	0.81(0.71-0.92)			0.87(0.70-1.09)	1.50(1.18-1.81)	1.20(1.03-1.41)	

Expendable income	> = £10	Ref***	Ref***	Ref***	Ref***	ns	Ref*	Ref***	Ref***
	£11-20	1.53(1.36-1.73)	1.50(1.34-1.67)	1.28(1.13-1.45)	1.45(1.27-1.65)		1.14(0.96-1.36)	1.32(1.16-1.50)	1.49(1.34-1.67)
	£21-30	2.04(1.70-2.44)	1.45(1.27-1.67)	1.23(1.05-1.44)	2.34(2.01-2.73)		1.17(0.94-1.46)	1.63(1.39-1.92)	1.82(1.58-2.10)
	>£30	2.94(2.47-3.49)	1.63(1.45-1.85)	1.55(1.36-1.77)	3.07(2.69-3.50)		1.35(1.13-1.63)	2.05(1.78-2.35)	2.43(2.14-2.76)

Hobby	No	Ref**	ns	Ref ***	ns	ns	ns	Ref***	Ref***
/Sport	Yes	1.17(1.05-1.30)		0.78(0.70-0.86)				0.77(0.68-0.86)	0.76(0.69-0.84)

Parents	No		Ref*	Ref***	Ref***	ns	Ref***	Ref***	Ref***
Provide	Yes		0.91(0.83-0.99)	0.44(0.39-0.48)	0.48(0.44-0.53)		0.37(0.31-0.43)	0.79(0.71-0.88)	0.59(0.54-0.65)

Peer	No		Ref***	Ref**	ns	Ref**	ns	Ref**	ns
Pressure	Yes		1.57(1.41-1.74)	1.20(1.05-1.32)		1.25(1.06-1.47)		0.81(0.72-0.92)	

Boredom	No		Ref***	Ref***	Ref***	Ref***	Ref***	Ref***	Ref***
	Yes		1.31(1.18-1.46)	3.72(3.34-4.15)	1.50(1.34-1.67)	1.94(1.65-2.27)	3.33(2.90-3.82)	2.68(2.39-3.00)	2.94(2.63-3.29)

Logistic regression techniques used a backwards conditional model. This method also controlled for local authority of school (n = 21). Local Authority AORs have not been displayed due to space but were significant for all outcome measures. ^§ ^drinking frequently = >1 session/week; drinking heavily = drink ≥5 drinks/session ≥1 per month. ^IMD = Index of multiple deprivation. AOR = Adjusted Odds Ratio. Ref = Reference category. 95% CI = 95% confidence intervals. ns = Not significant. Reported P values are * <0.05 ** <0.01 *** <0.001. Comparator groups for dependent variables in unsupervised consumption and access are, respectively, drinkers who do not: typically drink unsupervised inside; typically drink outside (in streets, parks and shops); buy their own alcohol; take alcohol from their parents without permission or; proxy purchase. For frequent drinking the reference group is drinkers who do not drink >1 session/week and for heavy drinking, drinkers who do not drink ≥5 drinks/session ≥1 per month.

Amongst those who drank alcohol, the most common method by which respondents' typical consumption fell outside the guidelines was through unsupervised inside consumption (57.4%; females, 61.3%; males, 53.0%), closely followed by heavy drinking (54.7%; females, 54.4%; males, 55.0%; Table 2). Female drinkers were more likely to report unsupervised inside consumption, as were those from the most affluent areas. The likelihood of unsupervised consumption inside was higher in those with an expendable weekly income over £10 with similar relationships seen for both drinking unsupervised outside and proxy purchase (Table 3). Increases in personal income were also associated with greater odds of self-purchase of alcohol and greater risks of frequent drinking and heavy drinking. Increasing age was associated with lower levels of proxy purchase but greater levels of unsupervised drinking inside, self-purchase, heavy and frequent drinking. Whilst females were more likely to take alcohol from parents without permission, they were less likely to proxy purchase, drink unsupervised outside, drink frequently or heavily (Table 3). Those living in the most affluent areas were more likely to take alcohol from parents without their consent and those in most deprived areas were at increased risk of proxy purchasing and frequent drinking. Involvement in a hobby/sport was protective against heavy and frequent drinking but for unsupervised drinking was only significantly associated with lower levels of outside drinking (Table 3).

Overall 49.6% of drinkers reported parental alcohol provision and this was especially associated with lower proportions of outside drinking, self and proxy purchase as well as less heavy and frequent drinking. Drinking due to peer pressure was significantly associated with higher unsupervised drinking inside and outside, and with taking alcohol from parents without permission. However, those stating peer pressure as a reason for drinking were less likely to drink frequently once confounding factors had been accounted for (Table 3). Boredom was associated with all categories of unsupervised drinking, and both frequent and heavy drinking (Table 3). Drinkers identifying any measure of unsupervised consumption or heavy or frequent drinking were significantly more likely to report alcohol related violence, regretted sex or forgetting things after drinking (Table 2). Frequent and heavy drinking behaviours were strongly associated with each other (Table 4). Further, those reporting any measure of unsupervised consumption were also more likely to drink frequently and to drink heavily. However drinkers reporting parental provision of alcohol were less likely to report both forms of excess drinking behaviours (Table 4).

**Table 4:** Proportion of youths drinking outside of national consumption guidance by reported consumption and access behaviours

		Excess drinking (%)			
Consumption and access		Frequent drinking		Heavy drinking	
Unsupervised inside drinking	Yes	26.2		65.7	
	No	17.2	<0.001	40.1	<0.001

Outside drinking (streets, parks, shops)	Yes	16.1		77.8	
	No	37.3	<0.001	45.1	<0.001

Parents provide	Yes	19.0		47.1	
	No	26.1	<0.001	63.3	<0.001

Take from parents	Yes	31.7		70.6	
	No	21.7	<0.001	53.8	<0.001

Proxy purchase	Yes	38.8		78.0	
	No	20.4	<0.001	52.2	<0.001

Self-purchase	Yes	39.9		80.1	
	No	16.6	<0.001	46.8	<0.001

Frequent drinking	Yes			90.1	
	No			44.6	<0.001

Heavy drinking	Yes	36.8			
	No	4.9	<0.001		

## Discussion

As with all cross-sectional and self-reporting surveys, there are limitations both to inference and extrapolation from results. Our measures of behaviours and negative outcomes relied on the honesty and accurate recollection of respondents. Further, sampling did not include individuals who were excluded from or had otherwise left school-based education, and deprivation was calculated on an ecological, not individual, basis. Adverse effects of alcohol were limited to three measures. These did not include correlates with prevalence of injury (e.g. hospital attendance or other potential consequences) or with wellbeing (e.g. effects on education, relationships). No quantitative measures of compliance could be collected from schools. Therefore, it was not possible to assess the extent to which selection bias may have affected the final sample. Further, results could not identify how specific findings are to the North West of England. Consequently, generalising results directly to wider populations should not be undertaken and any inferences on such groups should consider the variations in results associated with different demographic traits (see Table 3). Further, exploring a number of measures as proxies for the CMO guidance increases risks of Type I errors [24]. However, independent variables were limited to key demographics and factors previously identified as related to alcohol consumption [14,21]. Consequently, despite limitations, analyses presented here provide timely intelligence on potential cultural blocks to the implementation of the CMO guidance [4] through the provision of baseline data on young people's drinking behaviours eight months prior to the beginning of the guidance being implemented. Such data identify those risk and protective factors that can respectively be reduced and enhanced, and the possible public health benefits associated with successful implementation of the guidance.

Consistent with national surveys, alcohol consumption is a cultural norm across all deprivation strata by age 15 years [4] with higher levels in the most affluent groups (Tables 2). Changing such well-established norms will require prolonged interventions, targeted at those most at risk. Such public health interventions should ensure the effective provision of information on the potential benefits of abstinence or of complying with guidance on safer levels of consumption. Importantly for such interventions, our measures of compliance support its association with lower levels of acute alcohol related harms to young people (violence, regretted sex and recall issues; Table 2). For instance (amongst drinkers), 45.8% of those who have experienced alcohol related violence are frequent drinkers, compared with only 14.6% of those who have not experienced violence. However, social marketing campaigns that have now begun to use the English guidance (e.g. Why Let Drink Decide?[25,26]) face two major social norms issues. Firstly, drinking heavily is well established in children (Table 2). Whether this is with or without parental knowledge could not be identified here. Although parental supervision of such drinking may mitigate the risks of immediate harms (e.g. regretted sex and violence [20]), the risks to brain development are likely to remain unaltered [6]. The second norm amongst children is that unsupervised drinking within the home is routine, with such drinking at higher levels in the most affluent groups. This does not mean that it occurs without any advice from parents, as our proxy measure was drinking without parents in the home (Table 1). However, parental opportunities to monitor and potentially reduce heavy consumption will be limited while unsupervised consumption is common. In fact, of drinkers who identified that they drink at their own or a friend's home when parents are not there, around two thirds also drank heavily (Table 4). With a parental presence related to lower drinking levels in children, public health interventions may wish to adopt a social marketing approach targeting parents who allow children to drink when they are not at home and exploiting links with regretted sex, violence and memory lapses.

Relative alcohol affordability is also an important factor in promoting unsupervised, heavy and frequent alcohol use. While we did not measure the price of alcohol, those with a greater expendable income were more likely to buy their own alcohol and consume it at rates and in quantities outside of the guidance (Table 3), behaviours which are also associated with greater levels of alcohol related harms (Table 2). Although parental efforts to better understand expenditure by children or to reduce their personal income may help improve compliance with guidance, a minimum price per unit of alcohol would reduce access to the most affordable alcohol products they purchase and should be a public health priority [20,27].

Peer pressure is typically identified as an explanation for young people's drinking behaviours [28]. While we found drinking for such reasons positively associated with unsupervised drinking (both inside and outside) and taking alcohol from parents without permission, it was negatively associated with drinking frequently. While this needs further examination, it is consistent with heavier drinkers being group leaders in drinking behaviours rather than followers [29]. In general, however, boredom had a much stronger relationship with heavier, frequent and unsupervised drinking than peer pressure (Table 2 &3). With alcohol often available for under 15 pence per unit [20,30] for many young people drinking represents a cheap way of passing time with other forms of entertainment all too often geographically or financially inaccessible.

## Conclusions

Here, analyses of whether individuals' consumption of alcohol was within the CMO guidance and how behaviour falling outside of the guidance relates to suffering alcohol related harms were specific to the North West of England. The North West suffers some of the highest levels of alcohol problems in England [19], and the UK has some of the worst levels of alcohol misuse by youths in Europe [14]. However, internationally, misuse of alcohol by youths is already a major public health issue [31]. While many countries have adopted guidelines for adult drinking, very few have considered them for those under their legal age for purchasing alcohol. Our study suggests that in the absence of any guidance, unsupervised and heavy drinking have developed as social norms at least in parts of England. While the impact that guidance can have to reverse such norms and associated harms is currently poorly studied, national programmes to promote the CMO guidance across England, in combination with future iterations of this survey, should provide important longitudinal information on the effectiveness of the guidance.

In this cross-sectional study, likelihood of experiencing acute alcohol related harms was significantly lower in those 15-16 year olds surveyed who drank within the guidance than in those who drank heavily, frequently or who reported behaviours consistent with unsupervised or unguided drinking. With so many children reporting unsupervised and heavy drinking, long-term social marketing campaigns, educational interventions and parental support will be required to encourage better compliance with these aspects of the guidance. However, policy measures that establish a minimum unit price for alcohol and invest in alternatives to drinking that are attractive to young people could increase their chances of success and consequently help avoid much of the acute and long-term harm that alcohol already causes children.

## Competing interests

The authors declare that they have no competing interests.

## Authors' contributions

MAB analysed the data and produced the manuscript. MM, PC and SW assisted in production of the manuscript. MM assisted with data analysis. LS managed the survey. JD and KH contributed to data preparation, analysis and manuscript production. All authors read and approved the final manuscript.

## Pre-publication history

The pre-publication history for this paper can be accessed here:

http://www.biomedcentral.com/1471-2458/10/547/prepub

## References

[B1] Room R Babor T Rehm J Alcohol and public health Lancet 2005 365 519 530 15705462 10.1016/S0140-6736(05)17870-2

[B2] Jones L Bellis MA Dedman D Sumnall H Tocque K Alcohol-attributable fractions for England: alcohol-attributable mortality and hospital admissions 2008 Liverpool: North West Public Health Observatory, Centre for Public Health Research Directorate, Liverpool John Moores University

[B3] Strategy Unit Alcohol misuse: how much does it cost? 2003 London: Prime Minister's Strategy Unit

[B4] Donaldson L Guidance on the consumption of alcohol by children and young people 2009 London: Department of Health

[B5] Giedd JN The teen brain: insights from neuroimaging J Adolesc Health 2008 42 335 343 10.1016/j.jadohealth.2008.01.007 18346658

[B6] Guerri C Pascual M Mechanisms involved in the neurotoxic, cognitive, and neurobehavioural effects of alcohol consumption during adolescence Alcohol 2010 44 15 26 10.1016/j.alcohol.2009.10.003 20113871

[B7] Maldonaldo-Devincci AM Badanich KA Kirstein CL Alcohol during adolescence selectively alters immediate and long-term behaviour and neurochemistry Alcohol 2010 44 57 66 10.1016/j.alcohol.2009.09.035 20113874 PMC4199380

[B8] Squeglia LM Spadoni AD Infante MA Myers MG Tapert SP Initiating moderate to heavy alcohol use predicts changes in neuropsychological functioning for adolescent girls and boys Psychol Addict Behav 2009 23 715 722 10.1037/a0016516 20025379 PMC2802333

[B9] Fuller E (ed) Smoking, drinking and drug use among young people in England in 2008 2009 London: The Health and Social Care Information Centre

[B10] Zeigler DW Wang CC Yoast RA Dickinson BD McCaffree MA Robinowitz CB Sterling ML The neurocognitive effects of alcohol on adolescents and college students Prev Med 2005 40 23 32 10.1016/j.ypmed.2004.04.044 15530577

[B11] Rickards L Fox K Roberts C Fletcher L Goddard E Living in Britain. Results from the 2002 General Household Survey 2004 London: Office for National Statistics

[B12] Wells S Graham K Speechley M Koval J Drinking patterns, drinking contexts and alcohol-related aggression among late adolescent and young adult drinkers Addiction 2005 100 933 944 10.1111/j.1360-0443.2005.001121.x 15955009

[B13] Institute of Alcohol Studies Alcohol and the law 2009 St Ives: Institute of Alcohol Studies

[B14] Hibell B Guttormsson U Ahlstrom S Balakireva O Bjarnason T Kokkevi A Kraus L The 2007 ESPAD report: substance use among students in 35 European countries 2009 Stockholm: Swedish Council for Information on Alcohol and other Drugs

[B15] Higuchi S Matsuchita S Osaki Y Drinking practices, alcohol policy and prevention programmes in Japan Int J Drug Policy 2006 17 358 366 10.1016/j.drugpo.2006.05.005

[B16] Intoxicating Liquor Act 2003 2003 S.I. No. 362/2003

[B17] Ministry of Health, Welfare and Sport Opting for a healthy life. Public health policy in the Netherlands 2007-2010 2009 The Hague: Ministry of Health, Welfare and Sport

[B18] National Health and Medical Research Council Australian Guidelines to Reduce Health Risks from Drinking Alcohol 2009 Canberra: National Health and Medical Research Council

[B19] Deacon L Hughes S Tocque K Bellis MA Indications of public health in the English regions 8: alcohol 2007 York: Association of Public Health Observatories

[B20] Bellis MA Phillips-Howard PA Hughes K Hughes S Cook PA Morleo M Hannon K Smallthwaite L Jones L Teenage drinking, alcohol availability and pricing: a cross-sectional study of risk and protective factors for alcohol-related harms in school children BMC Public Health 2009 9 380 10.1186/1471-2458-9-380 19818118 PMC2770487

[B21] Bellis MA Hughes K Morleo M Tocque K Hughes S Allen T Harrison D Fe-Rodriguez E Predictors of risky alcohol consumption in schoolchildren and their implications for preventing alcohol-related harm Subst Abuse Treat Prev Policy 2007 2 15 10.1186/1747-597X-2-15 17493261 PMC1878473

[B22] Names and codes for super output area geography http://www.ons.gov.uk/about-statistics/geography/products/geog-products-area/names-codes/soa/index.html

[B23] Levin KA Currie C Inequalities in toothbrushing among adolescents in Scotland 1998-2006 Health Educ Res 2009 24 87 97 10.1093/her/cym096 18245045

[B24] Perneger TV What's wrong with Bonferroni adjustments Br Med J 1998 316 1236 1238 10.1136/bmj.316.7139.1236 PMC1112991 9553006

[B25] Government campaign asks - why let drink decide? http://www.dcsf.gov.uk/pns/DisplayPN.cgi?pn_id=2010_0016

[B26] Young people and alcohol http://www.direct.gov.uk/en/Parents/Yourchildshealthandsafety/Youngpeopleandalcohol/index.htm

[B27] Meier P Booth A O'Reilly D Stockwell T Sutton A Wilkinson A Wong R Independent review of the effects of alcohol pricing and promotion: Part B. Modelling the potential impact of pricing and promotion policies for alcohol in England: results from the Sheffield Alcohol Policy Model Version 2008 (1-1) 2008 Sheffield: Department of Economics, University of Sheffield

[B28] Donovan JE Adolescent alcohol initiation: a review of psychosocial risk factors J Adolesc Health 2004 35 e527 518 529 10.1016/j.jadohealth.2004.02.003 15581536

[B29] Mayeux L Sandstrom M Cillessen A Is being popular a risky proposition? J Res Adolesc 2008 18 49 74 10.1111/j.1532-7795.2008.00550.x

[B30] Ourlife Supermarket scandal: super-cheap alcohol sales in the North West. Report October 2009 2009 Manchester: Ourlife

[B31] Draft global strategy to reduce the harmful use of alcohol http://www.who.int/substance_abuse/alcstrategyaftereb.pdf 10.2471/BLT.19.241737 PMC7047030 32132758

